# Design and validation of an osteochondral bioreactor for the screening of treatments for osteoarthritis

**DOI:** 10.1007/s10544-018-0264-x

**Published:** 2018-02-14

**Authors:** Derek A. Nichols, Inderbir S. Sondh, Steven R. Litte, Paolo Zunino, Riccardo Gottardi

**Affiliations:** 1 Department of Mechanical Engineering and Materials Science, Pittsburgh, PA USA; 2 Department of Bioengineering, University of Pittsburgh, Pittsburgh, PA USA; 3 Department of Chemical Engineering, University of Pittsburgh, Pittsburgh, PA USA; 4 Department of Pharmaceutical Sciences, University of Pittsburgh, Pittsburgh, PA USA; 5 Department of Immunology, University of Pittsburgh, Pittsburgh, PA USA; 6 Department of Ophthalmology, University of Pittsburgh, Pittsburgh, PA USA; 7 the McGowan Institute for Regenerative Medicine, University of Pittsburgh, Pittsburgh, PA USA; 8 Department of Mathematics, Politecnico di Milano, Milan, Italy; 9 Department of Orthopedic Surgery, University of Pittsburgh, Pittsburgh, PA USA; 10 Ri.MED Foundation, Palermo, Italy

**Keywords:** Bioreactor, Osteochondral, Computational fluid dynamics, Composite tissues, Biphasic construct

## Abstract

Bioreactors are systems that can be used to monitor the response of tissues and cells to candidate drugs. Building on the experience developed in the creation of an osteochondral bioreactor, we have designed a new 3D printed system, which allows optical access to the cells throughout testing for in line monitoring. Because of the use of 3D printing, the fluidics could be developed in the third dimension, thus maintaining the footprint of a single well of a typical 96 well plate. This new design was optimized to achieve the maximum fluid transport through the central chamber, which corresponds to optimal nutrient or drug exposure. This optimization was achieved by altering each dimension of the bioreactor fluid path. A physical model for optimized drug exposure was then created and tested.

## Introduction

After initial screening on plated cells, assessment of new candidate pharmaceuticals primarily relies on animal and human testing; however, organoid models are being developed as a very relevant, complementary option as they can be employed for the medium to high throughput screening of drug candidates prior to costly animal or even human testing (Demircak and Arslan Yildiz n.d.; Sutherland et al. 2013). Furthermore, these *in vitro* microphysiological models (Wikswo 2014) are useful tools to dissect molecular pathways to identify disease mechanisms. However, their microfluidics are frequently constrained to one plane because of current manufacturing constraints, and this limits cell construct architecture to an environment that might not fully represent the complexity of native tissues, leading to inaccurate results (Neuži et al. 2012). 3D printing may help to overcome this limitation allowing for the creation of complex bioreactor geometries where more biomimetic tissue architectures can be hosted and media can move in three dimensions rather than merely two (Alexander et al. 2014). Thus, by increasing the complexity of the fluidics, more ways of directing cell exposure to candidate drugs are accessible. Additionally, tissue responses more representative of *in vivo* conditions (Bhattacharjee et al. 2016) prior or in parallel to animal testing can be utilized in order to decrease the need of animal use and improve the safety profile of the screened candidate drugs (Wikswo 2014). In fact, animal physiology is different than that of humans, which in itself already limits the predictive power of animal tests. Therefore, the use of human cells in a 3D environment that models human tissues could provide more physiologically relevant information of the effects of candidate drugs on human physiology before clinical trials (McManus 2013). Moreover, multiple “tissues” can be connected to study the effects of a drug on the target tissue but also verify possible side effects (Iannetti et al. 2016). Appropriate bioreactors for this purpose are then necessary, i.e., apparatuses in which to place and maintain native tissues or cells in a 3D scaffold environment whose response to a candidate drug can be monitored.

**Fig. 1:**
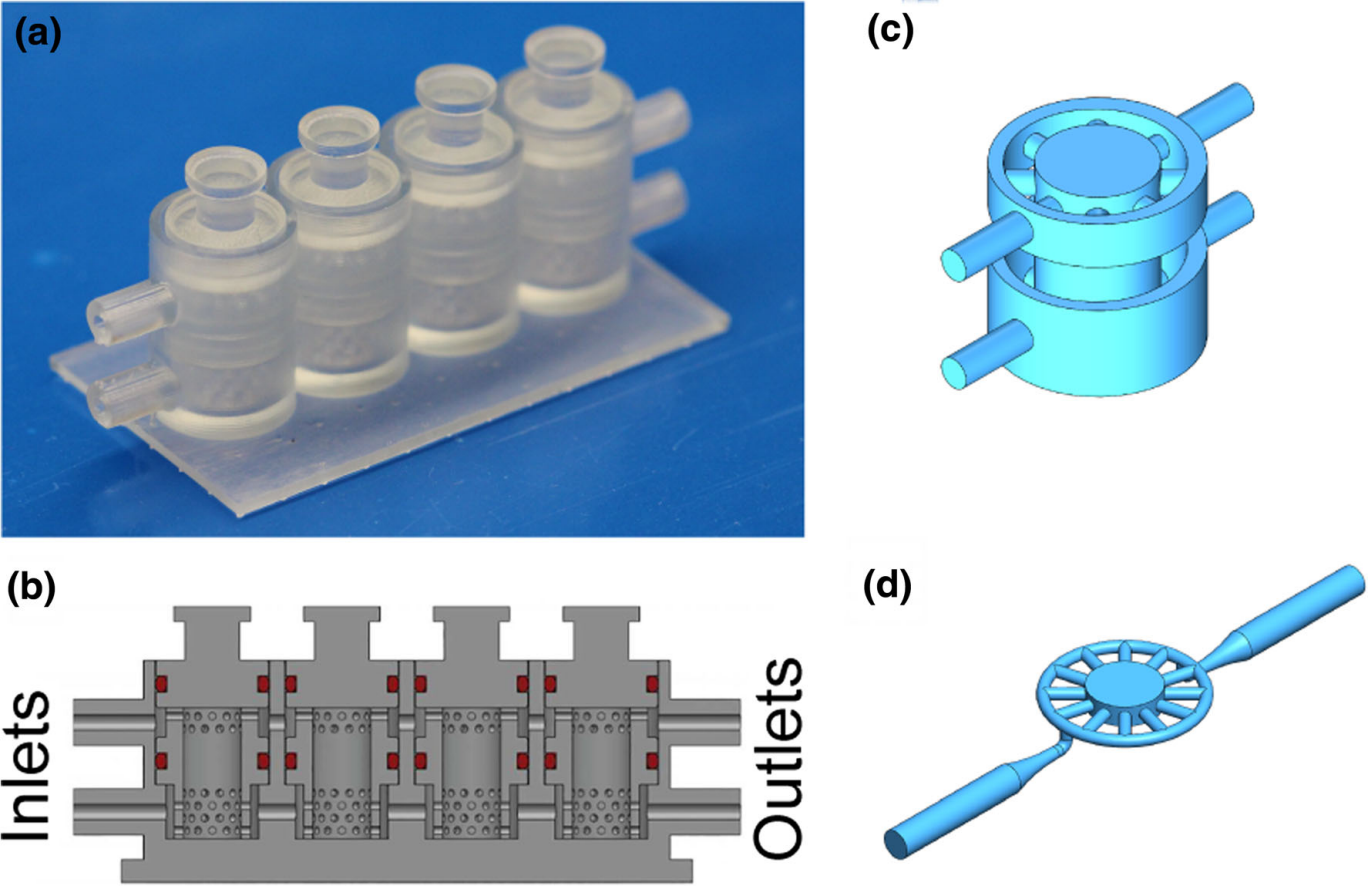
**a** Array of four bioreactors CAD models (Iannetti et al. 2016), (**b**) corresponding cross-section (Iannetti et al. 2016), (**c**) the fluid path (i.e. negative) of the current single bioreactor, (**d**) modified configuration of the bioreactor

In recent work, we have demonstrated how a bioreactor could be created to generate engineered biphasic osteochondral (OC) constructs comparable in size to native tissues and to culture native tissue over several weeks (Fig. 1a‑c) (Alexander et al. 2014; Iannetti et al. 2016; Lin et al. 2014). The middle cylinder of this bioreactor is where the cells under test are hosted in a scaffold, such as a porous polymer or a permeable hydrogel of methacrylated gelatin (GelMA).

This system was aimed at studying musculoskeletal diseases such as osteoarthritis (OA), which is characterized by the breakdown of cartilage lining the ends of long bones and currently has no cure (Lozito et al. 2013). When studying the mechanism of OA progression to identify possible therapies, it is crucial to consider both bone and cartilage simultaneously as there is growing evidence suggesting interplay between them (Alexander et al. 2014; Goldring and Goldring 2016; Lozito et al. 2013). While effective and innovative, the current OC microphysiological system relies on end point testing, rather than periodic and non-destructive assessment (Iannetti et al. 2016).

To grant optical access through the construct for continuous monitoring, the current bioreactor (Fig. 1a-c) has been modified by adding a glass coverslip over the 3D construct. For this purpose, the height of the chamber must be minimized to a height conducive to optical sectioning, and optical imaging tools such as fluorescently labeled cells or cells transfected with gene reporters must be employed (Grande and Bonfig 2015). This new system (Fig. 1d) is essentially a cross section of the previous bioreactor fluid path (Fig. 1c), which can be used for continuous optical monitoring.

A limitation of simply taking the cross section of the previous system, however, is that fluid flow through the central chamber is limited. Most of the media flows around the ring without ever making contact with the test cells in the middle. Indeed, resistance to flow through the central chamber is very high because of the relatively low permeability of the transparent hydrogel that would be used (such as GelMA). Thus, to change the fluid path and increase nutrients/drug exposure in the central chamber (Hsu et al. 2013a; Saleh 2002), we created an improved bioreactor model by 3D printing. First, we tested and optimized the internal fluid path via computational fluid dynamics (CFD) with the ANSYS CFX commercial software. After developing a computationally guided analysis protocol, we set up an optimization procedure of the bioreactor design parameters within our experimental constraints. Finally, we compared the performance of the optimal shape against the simple ring model, detecting significant improvements that were confirmed experimentally with 3D printed bioreactors models.

## Methods

### Designing the flow path

Models of the flow path were created using the CAD software SOLIDWORKS 2016 (Waltham, MA). The design constraints of the bioreactor were the following: (i) the volume of the central chamber must be roughly 10 μL; (ii) the height of the central chamber must not limit optical sectioning via imaging; (iii) the overall dimensions must allow for the design to fit into a 96-well plate unit well. To allow for optical sectioning via a confocal microscopy, the height of the cell housing was kept to 1 mm (Grande and Bonfig 2015) consequently requiring the diameter of the central chamber to be 3.6 mm for a volume of ~10 μL. Hence, to fit into a 96-well plate, the surrounding ring must be no larger than 6.86 mm in diameter. Given those design constraints, the other parameters varied within an optimization procedure were: (A) the diameter of the surrounding channel, (B) the step height, (C) the outer ring diameter, (D) the pore diameter, and (E) the number of pores (see Fig. 3 for a visualization of these). These features were changed independently to observe how each affected the flow in the entire model. Designs were saved in SOLIDWORKS as IGES files that were passed to the CFD analysis software described in the next section.

### Computational fluid dynamics modelling

The CFD analysis software ANSYS Fluid Flow (CFX) v. 15.0 (Canonsburg, PA) was used for all simulations. The simulation setup was similar the one previously developed by our research team (Iannetti et al. 2016), in which a dual fluidic bioreactor was used for high throughput screening. The IGES geometry files were imported into the project, and the chamber hosting the cells (central chamber) was represented as a porous domain, conferring it the properties of a permeable solid that permits fluid flow. Two different hydrogels were used in the simulations: a photocrosslinked methacrylated gelatin (GelMA) and agarose, the properties of each are reported in Table 1 (Johnson and Deen 1996; Taffetani et al. 2014).

**Table 1:** Properties of the material residing in the central chamber (Johnson and Deen 1996; Taffetani et al. 2014)

Material	Porosity	Permeability (m^2^)	Molar Mass (kg/kmol)	Density (kg/m^3^)
GelMA	0.8	1*10^−16^	1.00117	1190
Agarose	0.985	6.16*10^−16^	630.549	1026

A volume flow rate of 1 mL/day was imposed at the inlet, and the outlet was open to the environment. Steady state velocities through the central chamber resulting from the ANSYS simulation were measured in CFX Post and plotted against each specific change in the bioreactor geometry to determine the relationships between design features and central velocity. The assessment of each model was based on the velocity of the fluid through the middle of the central chamber, which gives a representation of nutrients/drug exposure.

A transient simulation was also set up in which the central chamber was filled with the porous material while the remaining bioreactor and chamber were filled with air. The simulation was then run with fluid starting from the inlet to see how the central chamber initially reacts to the onset of flow and to observe how long it takes for the porous central chamber to reach steady-state conditions.

### 3D printing

The bioreactor was printed by stereolithography (SLA) using a 3Dsystems Viper si2 (Rock Hill, SC) printer and Somos WaterShed XC 11122 (Elgin, IL) resin. The resolution of the printer is 50 μm and the smallest possible printable void is 600 μm.

### Fluidic validation

Agarose (2-Hydroxyethylagarose Type VII, low gelling temperature, Sigma-Aldrich) was mixed with water at a concentration of 2% *w*/*v* and heated on a hot plate until transparent to ensure full dissolution. The hot agarose solution was poured in a mold and allowed to rapidly cool to gel. Methacrylated gelatin (GelMA) was prepared as previously described (Lin et al. 2014). Briefly, dry GelMA powder was resuspended in PBS, mixed with the previously described LAP (photoinitiatior) (Lin et al. 2014) at a final concentration of 10% GelMA and 0.15% LAP. This solution was poured into a mold and cured for 1.5 min using an UV light with wavelength of 390–395 nm.

Once the bioreactor was printed and assembled, the central chamber was filled with the GelMA or agarose scaffold. The inlet was connected to a syringe filled with water placed in a Kiyatec FC230 (Greenville, SC) pump which forced the water through the bioreactor at a rate of 1 mL/day. Food coloring was added to the water in order to better observe the flow through the central chamber. Pictures were then taken at 15-min intervals to observe the flow of the fluid through the bioreactor.

## Results

### Bioreactor design

**Fig. 2:**
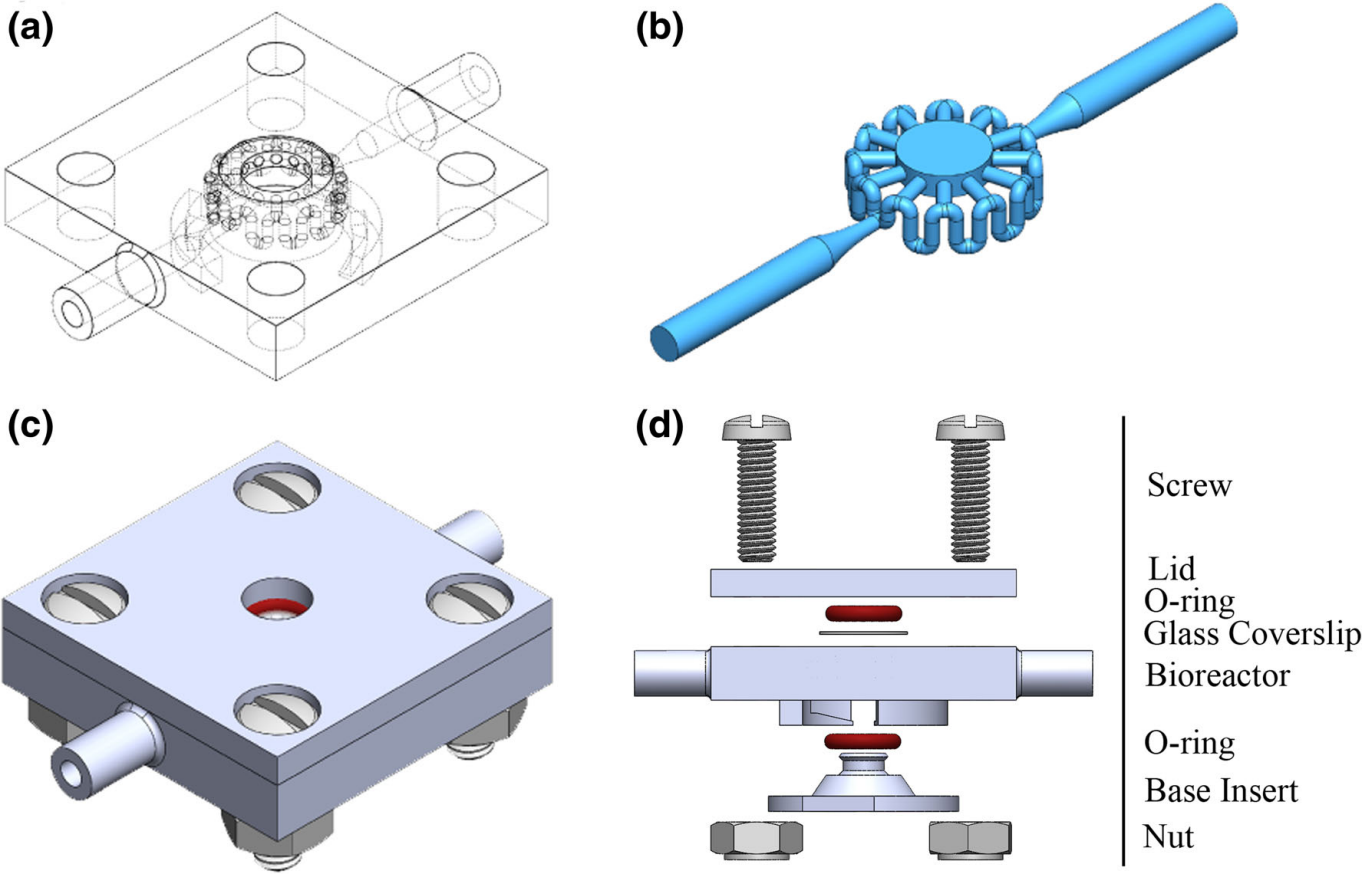
**a** Bioreactor, (**b**) volume occupied by fluid and constructs, (**c**) assembled bioreactor model, (**d**) exploded view

The main constraint for the new bioreactor design was to create a prototype that granted optical access to the cells throughout the central chamber for the duration of the experiment. In order to allow for optical access to the cells, the thickness of the cell construct could not exceed 1 mm in height as this is the typical range of the maximum image sectioning depth of a standard confocal microscope with a 10× objective (Grande and Bonfig 2015). The main block of the bioreactor (Fig. 2a) contains the fluidics feeding the central chamber (Fig. 2b) that is sealed with a removable base and lid with a coverslip (Fig. 2c-d).

In the bioreactor design, before shape optimization (Fig. 1c), cells were hosted in the central chamber within a thick scaffold, which did not allow optical access to most of the construct volume (Lozito et al. 2013). The specific goal for this research was to define the optimal fluidic design of an individual bioreactor chamber, such as the one seen in Fig. 2b, that allows complete permeation of the engineered constructs with nutrients and other soluble factors. The flow was controlled by changing the channel’s geometry to tune local pressure differences, and the effectiveness of each design option was quantified measuring the velocity of the fluid through the central chamber. In fact, since the flow rate at the inlet is fixed, the higher the velocity through the central chamber, the greater the total amount of mass transport and consequent drug and nutrient exposure for the cells over time.

### Design optimization

**Fig. 3:**
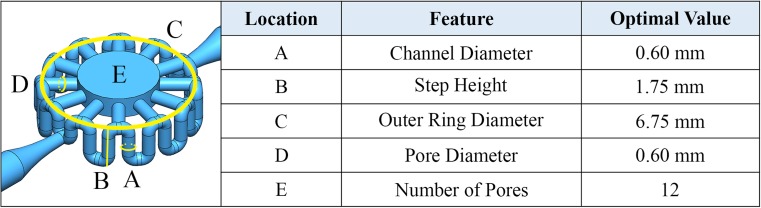
Defining the features of the bioreactor

Optimization of the bioreactor to control the flow through the inner chamber has been achieved by altering the dimensions of the features reported in Fig. 3.

**Fig. 4:**
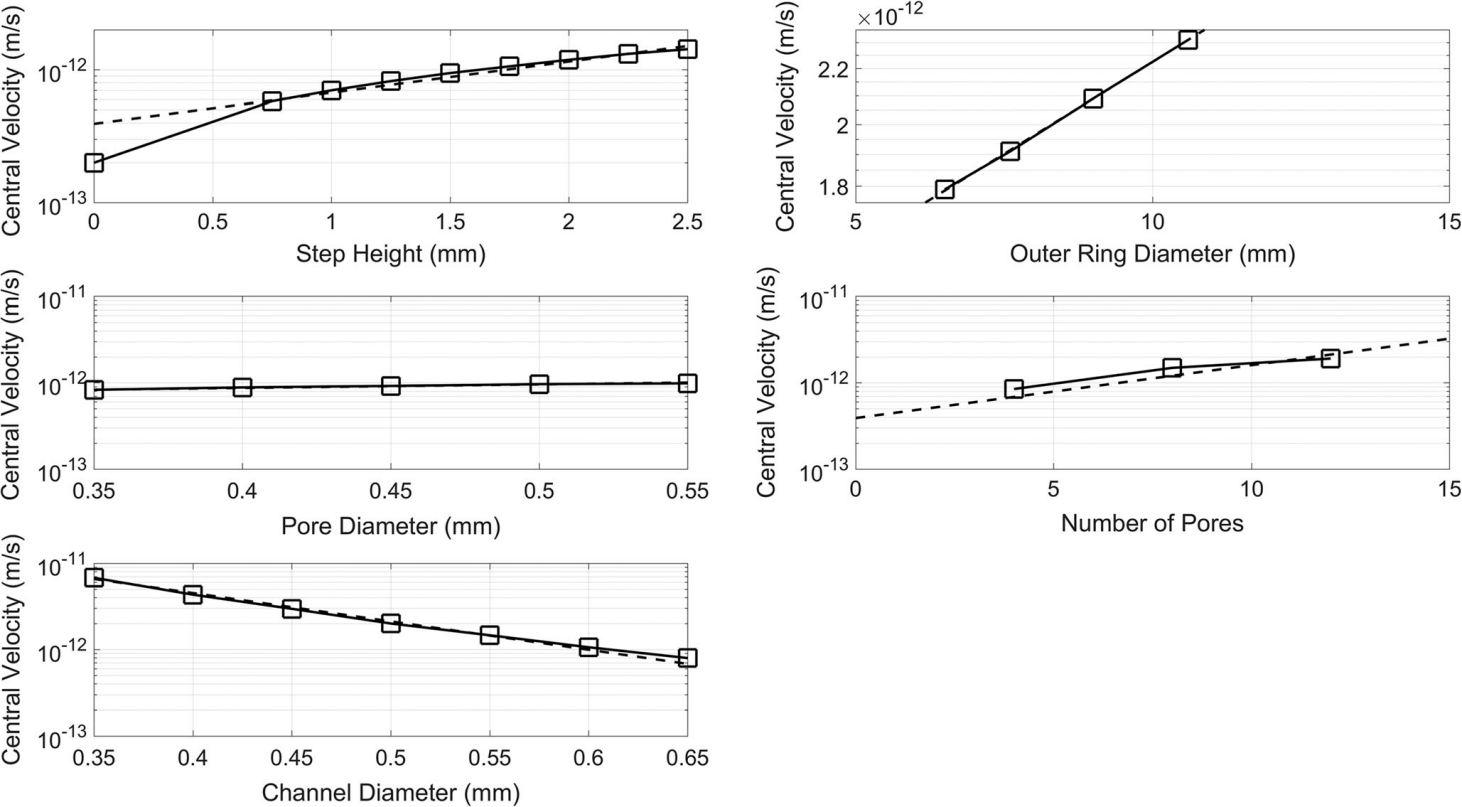
Plots of the central velocity versus various dimensions of features in Fig. 3

Each dimension has been progressively increased in 0.05 mm increments while all other dimensions were kept constant, and the flow velocity in the central chamber has been plotted against the respective dimension cumulative increments (Fig. 4). A line plot was used so that local changes in the velocity from one configuration to the next can be visualized. Furthermore, the dashed line represents the regression of the data obtained by a simple exponential y = a*exp.(bx). The exponent *b* can be seen as an indicator of the sensitivity of the central velocity with respect to the geometric parameter being changed, while *a* is just a scaling constant. The values for *b* are −7.565 for channel diameter, 0.5387 for step height, 0.06268 for outer ring diameter, 0.9462 for pore diameter, and 0.1412 for the number of pores. The higher the exponent, the more sensitive the velocity from the corresponding input parameter.

When plotted on a log scale, fluid velocity is directly dependent on the step height, outer ring diameter, pore diameter, and number of pores whereas the correlation with the channel diameter is inversely dependent. Once parameter constraints are defined, it is possible to identify the maximum flux through the central chamber hosting the engineered constructs simply using these empirical relationships for each design feature.

### Design optimization

The step height can be extended only a certain amount before it runs into other portions of the model; therefore, the maximum size for the step height is 1.75 mm. To eventually be used in a 96-well plate, the entire ring of the design must fit within a 6.8 mm diameter circle. The pore diameter can only be as large as the channel diameter, and, because the magnitude of the exponential term *b* (our indicator of sensitivity) is much larger for the channel diameter relationships than that of the pore diameter, it was determined that the model benefits more from a small channel than large pores. To satisfy these requirements and taking the 3D printing capabilities into account, the optimal dimensions were determined and are reported in Fig. 3.

### Design comparison

Taking advantage of the possibility offered by 3D printing to exploit the third dimension to develop the fluidic path, an optimized bioreactor design was created, and its steady-state ANSYS fluid flow results were compared to a simple circular ring model to determine how much the design optimization improved mass transport. The steady-state simulation results produced a central velocity of 5.026e-13 m/s for the ring model and 1.409e-12 m/s for the step model, with an almost three hundred percent increase for the latter.

Much more computationally demanding transient simulations were also performed, starting with the central chamber filled with a porous medium while the rest of the bioreactor was filled with air. The results show that the central chamber reaches steady-state conditions upon the fluids first pass around the surrounding channel. This takes ~2000 s for the simple ring model compared to ~3000 s for the step model; however, this time increase takes place simply because the step model has a longer path to travel until the flow moves completely around the surrounding channel to the outlet. For the simulation with the central chamber filled with agarose, the mass flow rate across a transverse plane placed at the midpoint of the chamber was measured. For the ring model, the mass flow was 4.640e-16 kg/s while the step model facilitated a mass flow of 1.099e-15 kg/s through the central chamber. Thus, at steady-state the step model produced a net mass flow rate 237% that of the ring model. This greater mass flux outweighs the fact that the step model chamber takes 50% longer to reach steady state as the volume flowing through the central chamber is 2.37 times greater.

**Fig. 5:**
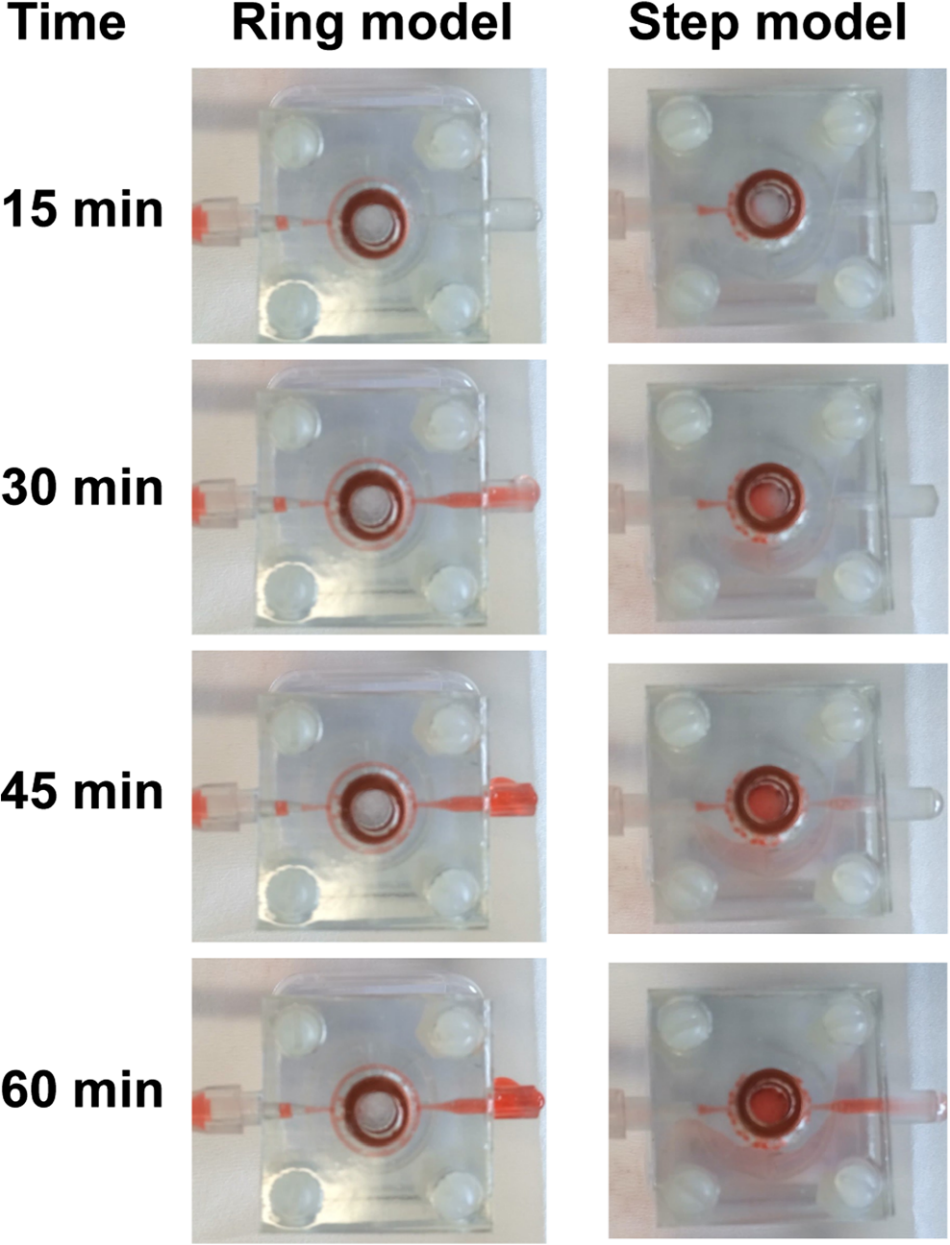
Results from laboratory testing proving that the step model attains more drug exposure through the central chamber

These two models were 3D printed and tested experimentally by flowing dyed water through the systems at a rate of 1 mL/day and making observations every 15 min (Fig. 5). The results from the laboratory tests confirm the simulation results. It is apparent that the step bioreactor achieves more nutrients/drug exposure than the ring bioreactor as evidenced by more volume of dye flowing through the central chamber over the same time period.

## Discussion

As shown in the previous section, the flux through the central chamber can be controlled by simply altering the geometry of the device. Experimental data corresponding to the simulations confirmed that, by using the ANSYS simulations as a guide, the design could be optimized to significantly increase the flux through the central chamber while still leaving an exit path for any air bubbles. The results indicate that maximization of fluid velocity, and therefore the total nutrients/drug exposure, can be achieved by minimizing the channel diameter and maximizing all other design features; therefore, the sole constraints are determined by the resolution of the 3D printer and by the overall design considerations of the model. With this optimized bioreactor, the central chamber was filled with either GelMA or agarose, each of which affects the results differently because of their different permeabilities. In particular, 2% agarose had a higher permeability than GelMA, which in turn allows greater nutrients/drug exposure of the cells. Furthermore, the transient simulations show that both configurations have reached steady-state approximately around the 45-min image as shown experimentally in Fig. 5. Consequently, at this time, the fluid is flowing through the central chamber at its maximum speed, which confirms the greater effectiveness for the optimized design bioreactor.

The method of increasing the path length to increase drug exposure was seen in work by Hsu et al. (Hsu et al. 2013b); however, this work only exploits fluidics in two dimensions which greatly increases the bioreactor footprint and makes the method impractical when utilizing a 96-well plate format. With the use of 3D printing, this extra pathlength can be moved to the third dimension which increases the pathlength without changing the footprint. This increased pathlength effectively increases the system’s hydraulic resistance of the surrounding channel, thus leading to more fluid to flow through the central chamber whose resistance does not change. Higher hydraulic resistance could also in principle be achieved by decreasing the channel diameter (Idel’chik 1966; Saleh 2002), however this option is currently limited by the resolution of the 3D printer. Hydraulic resistance plays a key role in microfluidics as the small channel diameters produce a large impact on the resistance of the fluid path. The effect of hydraulic resistance has been for instance explored in the experimental study on flow-resistance law for small diameter plastic pipes (Bagarello et al. 1995) which agrees well with our results. Better control on 3D printing resolution at the micrometer scale could then greatly benefit the development of 3D printed microfluidic systems with reduced footprint.

A major limitation in this study arises from the fact that devices were printed close to the printer’s tolerances. The resulting fluid path, although designed as perfectly circular, likely has a rough step profile at such a small scale, and this imperfection might affect the flow in a way that is not entirely known. Mitigating this uncontrolled effect, both circular and step bioreactor design are likely to be similarly affected, thus the relative difference observed in the experimental outcomes should be reliable and confirm the differences between the models’ findings.

**Fig. 6:**
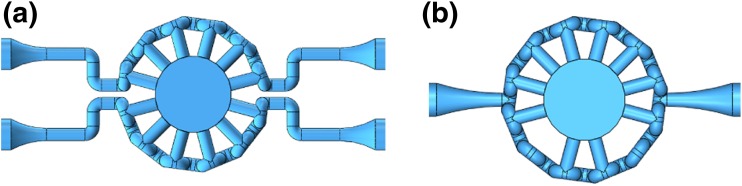
**a** Dual inlet bioreactor, (**b**) asymmetric bioreactor

Having confirmed the predictive value of the simulations, different and unique models can be created and tested in ANSYS to account for specific experimental needs in engineering multi-phase and multi-component constructs or for delivering drug candidates to specific construct locations. For instance, a dual inlet model (Fig. 6a) can be used to engineer bone on one side of the central chamber with cartilage on the other. Similarly, a compound could be delivered to only one tissue to study cartilage-bone interaction, when only one of the two is subject to a stress signal or to a drug. Alternatively, an asymmetric, single-inlet bioreactor (Fig. 6b) can be designed to test two different drug exposures simultaneously. This can be accomplished by changing the number of pores on the two sides of the bioreactor or by changing the distribution of the GelMA or agarose in the central chamber. By utilizing two different concentrations in differing configurations, differing drug exposures can be achieved in different areas of the 3D construct.

## Conclusions

A computationally-guided bioreactor was successfully designed to increase perfusion of cells in a construct. As predicted by the simulations and confirmed experimentally, compared to the circular ring model, the optimized step bioreactor achieves 2.37 times greater flux through the central chamber. The optimized bioreactor has multiple advantages: (1) it maximizes nutrients/drug exposure to the cells under test, (2) it minimizes the dimensions of the model, (3) it allows for optical access within the 3D construct, and (4) it maintains dimensions compatible with a standard 96-well plate.

With the optimization procedure validated, different and more complex bioreactor configurations can be created. For instance, true OC tests can be conducted on both bone and cartilage simultaneously by utilizing the dual inlet bioreactor introduced in section 4. An array of bioreactors can also be simulated to study how the pressure-drop changes through each subsequent model. Based on these studies, a bioreactor comprised of an array of identical units as the ones described above, is currently being implemented to allow for medium to high throughput *in vitro* drug screening.

## Abbreviations

3D: Three dimensional
OA: osteoarthritis
OC: osteochondral
GelMA: methacrylated gelatin
